# ID 312 - Anticoagulação Oral na Fibrilação Atrial: revisão sistemática e metanálise em rede de ensaios clínicos randomizados

**DOI:** 10.5327/2237-9622.2025.v34s1.312

**Published:** 2025-11-25

**Authors:** Suelem Tavares da Silva Penteado, Isabella Souza do Valle, Fabiana Rossi Varallo, Leonardo Regis Leira Pereira

**Keywords:** anticoagulantes de ação direta, eficácia, segurança, varfarina

## Abstract

**Introdução:**

A fibrilação atrial (FA) afeta cerca de 1,5 milhão de brasileiros, destacando-se como causa significativa de hospitalizações cardiovasculares. O uso de anticoagulantes é crucial na prevenção de eventos tromboembólicos, embora se associe a riscos de sangramento, especialmente em pacientes com fatores de risco adicionais. Anticoagulantes orais diretos (DOACs – do inglês, ) têm sido preferidos devido a seu perfil de segurança e eficácia, porém não foram incorporados ao Sistema Único de Saúde (SUS). Nesse sentido, avaliar a eficácia e a segurança dos DOACs em comparação à varfarina nas políticas de saúde estaduais torna-se essencial para garantir o acesso racional e eficaz aos tratamentos para FA. Sendo assim, o objetivo foi sintetizar as evidências sobre o uso de DOAC em pacientes com FA e avaliar sua eficácia e segurança por meio de revisão sistemática e metanálise em rede.

**Método:**

Uma revisão sistemática (RS) de ensaios clínicos randomizados (ECR) foi conduzida, identificados a partir das estratégias de busca elaboradas nas bases de dados, que avaliassem a eficácia e a segurança dos DOACs em comparação à varfarina em pacientes adultos com FA. O processo de elegibilidade consistiu na triagem dos estudos para avaliação de título e resumo, utilizando-se a plataforma Rayyan®; posteriormente, foi realizada uma avaliação por texto completo. Para avaliação do desfecho de eficácia, foram coletados os desfechos de acidente vascular cerebral (AVC) isquêmico e hemorrágico, infarto agudo do miocárdio (IAM), tromboembolismo, mortalidade geral e cardiovascular; para avaliação da segurança, foram coletados os desfechos de hemorragia intracraniana, sangramento maior, sangramento clinicamente relevante e risco de sangramento gastrointestinal. Foram realizadas análise de risco de viés pela metodologia Rob 2.0 e análise da certeza da evidência pela metodologia GRADE (do inglês, ). Para a metanálise em rede, foram utilizados os softwares NMAstudio® e MetaInsight®, e análise SUCRA (do inglês, ) e de ranqueamento também foi realizada.

**Resultados:**

Foram incluídos quatro ECR em nossa RS, sendo apenas um estudo sem cegamento. Os DOACs reduziram AVC, especialmente hemorrágico, tromboembolismo, mortalidade geral e cardiovascular e hemorragia intracraniana, em comparação à varfarina. Apesar disso, foi observado elevado risco de viés para o estudo de RE-LY que avaliou dabigatrana. A partir das metanálises em rede. foi identificado que rivaroxabana e dabigatrana na apresentação de 150 mg foram os DOACs com melhores resultados para os desfechos de eficácia; e edoxabana com melhor relação acidente vascular encefalíco hemorrágico e sangramento maior.

**Conclusão:**

Apesar dos estudos que avaliam eficácia e segurança de DOACs serem de não inferioridade destes em comparação à varfarina, é possível observar alguma diferença em relação aos desfechos ao risco de AVC, especialmente o AVC hemorrágico e tromboembolismo. Além disso, os DOACs apresentaram resultados positivos em relação aos desfechos de mortalidade por qualquer causa e mortalidade cardiovascular e reduziram substancialmente o desfecho de hemorragia intracraniana, que é uma complicação importante e, muitas vezes, fatal no uso de anticoagulantes. A decisão sobre o DOAC mais apropriado deve, em última análise, ser individualizada e baseada numa tomada de decisão partilhada que reflita os benefícios, os riscos, as contraindicações, os custos e as preferências do paciente relacionados com o tratamento.

